# Expression of the BRCA1 complex member BRE predicts disease free survival in breast cancer

**DOI:** 10.1007/s10549-012-2122-5

**Published:** 2012-06-16

**Authors:** Sylvie M. Noordermeer, Marloes Wennemers, Saskia M. Bergevoet, Adrian van der Heijden, Evelyn Tönnissen, Fred C. G. J. Sweep, Joop H. Jansen, Paul N. Span, Bert A. van der Reijden

**Affiliations:** 1 Department of Laboratory Medicine, Laboratory of Hematology, Radboud University Nijmegen Medical Centre, Nijmegen Centre for Molecular Life Sciences, Geert Grooteplein Zuid 8, 6525 GA Nijmegen, The Netherlands; 2 Department of Radiation Oncology, Radboud University Nijmegen Medical Centre, Geert Grooteplein Zuid 8, 6525 GA Nijmegen, The Netherlands; 3 Department of Laboratory Medicine, Radboud University Nijmegen Medical Centre, Geert Grooteplein Zuid 32, 6525 GA Nijmegen The Netherlands

**Keywords:** BRE, Radiotherapy, DNA damage repair, BRCA1, Breast cancer

## Abstract

Breast cancer is one of the leading causes of cancer mortality in women. Recent advances in gene expression profiling have indicated that breast cancer is a heterogeneous disease and the current prognostication using clinico-pathological features is not sufficient to fully predict therapy response and disease outcome. In this retrospective study, we show that expression levels of *BRE*, which encodes a member of the BRCA1 DNA damage repair complex, predicted disease-free survival (DFS) in non-familial breast cancer patients. The predictive value of *BRE* expression depended on whether patients received radiotherapy as a part of their primary treatment. In radiotherapy-treated patients, high *BRE* expression predicted a favorable DFS (hazard ratio (HR) = 0.47, 95 % confidence interval (CI) = 0.28–0.78, *p* = 0.004), while in non-treated patients, high *BRE* expression predicted an adverse prognosis (HR = 2.59, 95 % CI = 1.00–6.75, *p* = 0.05). Among radiotherapy-treated patients, the prognostic impact of *BRE* expression was confined to patients with smaller tumors (HR = 0.23, 95 % CI = 0.068–0.75, *p* = 0.015) and it remained an independent factor after correction for the other prognostic factors age, tumor size, lymph node involvement, and histological grade (HR = 0.50, CI = 0.27–0.90, *p* = 0.021). In addition, high *BRE* expression predicted a favorable relapse-free survival in a publicly available dataset of 2,324 breast cancer patients (HR = 0.59, CI = 0.51–0.68, *p* < 0.001). These data indicate that BRE is an interesting candidate for future functional studies aimed at developing targeted therapies.

## Introduction

Despite great improvements in diagnostic imaging techniques and treatment, breast cancer remains one of the leading causes of cancer mortality in women. Prognostication of breast cancer patients nowadays relies highly on classical clinico-pathological features, such as tumor size, histological grade, age, and lymph node metastases [1]. However, it remains a challenge to accurately predict disease outcome based on these parameters, which is necessary not to under or over treat the patients.

Over the last 20 years, there has been great interest in developing prognostic patient classification methods based on molecular screenings. Genome-wide gene expression screens have identified expression profiles that predict disease outcome and therapy response. For example, in several large patient studies, a 70-gene signature called the “MammaPrint” (Agendia, Amsterdam, the Netherlands) has been shown to outperform classical prognostication methods [2–4]. Together with other molecular classification methods [5, 6], these data indicate that the identification of differential gene expression has great potential for improved prediction of disease outcome and subsequent treatment decisions.

DNA double strand breaks (DSBs) are one of the most cytotoxic types of DNA damage. The importance of proper repair of these breaks to maintain genomic integrity is exemplified by recurrent mutations in genes involved in DSB repair in various cancers. For example, *BRCA1* mutations occur in approximately 20 % of familial breast cancer cases [7–9]. The importance of the BRCA1 multi-protein complex has been exemplified by the identification of polymorphisms and haplotypes within other BRCA1 complex members, such as *RAP80* and *ABRAXAS*, both in *BRCA1/2* mutated and non-mutated familial breast cancer patients. However, the clinical impact of these polymorphisms remains to be confirmed [10–15]. Furthermore, *BRCA1* expression levels seem to predict breast cancer outcome in non-familial cases [16–19] although data are not consistent [20].

Recently, it has been shown that high expression of *BRE* (Brain and Reproductive organ-Expressed), another member of the BRCA1 complex [21–24], denotes a favorable prognosis in acute myeloid leukemia (AML) [25–27]. In this study, we demonstrate that *BRE* expression levels in breast cancer tumor tissue contained prognostic information in a cohort of 229 non-familial breast cancer patients, establishing the relevance of this DNA damage repair factor in breast cancer.

## Materials and methods

### Breast cancer samples

Frozen breast cancer tissue sections were available for two independent cohorts of 229 patients in total who had undergone resection of their primary tumor, as described before [28–30]. Patients underwent surgical resection of their primary tumor between November 1987 and December 1997 and were selected by the availability of RNA samples in the tumor bank of the Department of Chemical Endocrinology of the Radboud University Nijmegen Medical Centre (Nijmegen, The Netherlands). This bank contains tumor material from five different hospitals of the Comprehensive Cancer Centre East in the Netherlands. Patients had no previous diagnosis of carcinoma, no distant metastases at time of diagnosis, and no evidence of disease within 1 month after primary surgery. Patients that received neoadjuvant therapy or were diagnosed with carcinoma in situ were excluded from this study. Patients were treated with protocols established at that time. 60 % of the patients underwent mastectomy (137/229) and the remaining patients underwent lumpectomy. 74 % of the patients (169/229) received radiotherapy following surgery and 39 % (90/229) received systemic adjuvant treatment, in combination with radiotherapy or not. Adjuvant treatment consisted of endocrine treatment with tamoxifen and/or chemotherapy. Detailed patient characteristics can be found in Table 1. The median follow-up period of censored patients was 107.5 months. This study was performed according to REMARK guidelines [31].

**Table 1:** Clinico-pathological characteristics of 229 non-familial breast cancer patients

	Total cohort					Non-radiotherapy-treated					Radiotherapy-treated				
	Low *BRE* ^a^ (*N* = 115)		High *BRE* ^a^ (*N* = 114^b^)		*p*	Low *BRE* ^a^ (*N* = 32)		High *BRE* ^a^ (*N* = 27^b^)		*p*	Low *BRE* ^a^ (*N* = 83)		High *BRE* ^a^ (*N* = 86^b^)		*p*
Age (*N* = 229), mean (range)	59.9	(31–88)	59.2	(32–86)	0.775^d^	63.0	(33–88)	61.5	(35–86)	0.784^d^	58.7	(31–85)	58.3	(32–83)	0.816^d^
Menopausal status (*N* = 229)					1.000^e^					1.000^e^					1.000^e^
Premenopausal, no. (%)	29	(25.2)	28	(24.6)		6	(18.8)	5	(18.5)		23	(27.7)	23	(26.7)	
Postmenopausal, no. (%)	86	(74.8)	86	(75.4)		26	(81.3)	22	(81.5)		60	(72.3)	63	(73.3)	
Nodal category (*N* = 208)					0.923^e^					0.310^e^					0.806^e^
Negative, no. (%)	61	(58.1)	59	(57.3)		25	(86.2)	18	(72.0)		36	(47.4)	41	(52.6)	
1–3 involved lymph nodes, no. (%)	29	(27.6)	31	(30.1)		4	(13.8)	7	(28.0)		25	(32.9)	24	(30.8)	
≥4 involved lymph nodes, no. (%)	15	(14.3)	13	(12.6)		0	(0)	0	(0)		15	(19.7)	13	(16.7)	
Radiotherapy (*N* = 228)					0.547^e^					NA					NA
Treated, no. (%)	83	(72.2)	86	(76.1)		0	(0)	0	(0)		83	(100)	86	(100)	
Non-treated, no. (%)	32	(27.8)	27	(23.9)		32	(100)	27	(100)		0	(0)	0	(0)	
Surgery (*N* = 229)					0.282^e^					0.495^f^					0.219^f^
Mastectomy, no. (%)	73	(63.5)	64	(56.1)		30	(93.8)	27	(100)		43	(51.8)	36	(41.9)	
Lumpectomy, no. (%)	42	(36.5)	50	(43.9)		2	(6.3)	0	(0)		40	(48.2)	50	(58.1)	
Adjuvant systemic therapy (*N* = 228)					0.230^f^					0.537^f^					0.269^f^
None, no. (%)	70	(60.9)	68	(60.2)		26	(81.3)	18	(66.7)		44	(53.0)	50	(58.1)	
Endocrine therapy, no. (%)	30	(26.1)	28	(24.8)		3	(9.4)	4	(14.8)		27	(32.5)	24	(27.9)	
Chemotherapy, no. (%)	13	(11.3)	9	(8.0)		2	(6.3)	2	(7.4)		11	(13.3)	7	(8.1)	
Endocrine + Chemotherapy, no. (%)	2	(1.7)	8	(7.1)		1	(3.1)	3	(11.1)		1	(1.2)	5	(5.8)	
Histology grade (*N* = 168)					0.406^e^					0.151^f^					0.123^e^
I, no. (%)	9	(10.5)	4	(4.9)		1	(4.3)	1	(5.3)		8	(12.7)	3	(4.8)	
II, no. (%)	36	(41.9)	35	(42.7)		6	(26.1)	10	(52.6)		30	(47.6)	25	(39.7)	
III, no. (%)	41	(47.7)	43	(52.4)		16	(69.6)	8	(42.1)		25	(39.7)	35	(55.6)	
Tumor type (*N* = 193)					0.744^e^					0.721^f^					1.000^e^
Ductal, no. (%)	73	(73.0)	70	(75.3)		22	(71.0)	18	(81.8)		51	(73.9)	52	(73.2)	
Lubular, no. (%)	15	(15.0)	15	(16.1)		3	(9.7)	2	(9.1)		12	(17.4)	13	(18.3)	
Other (mixed/unknown), no. (%)	12	(12.0)	8	(8.6)		6	(19.3)	2	(9.1)		6	(8.7)	6	(8.5)	
Tumor size (*N* = 227)^c^					0.014^f^					0.853^f^					0.005^e^
pT1, no. (%)	33	(28.7)	50	(44.6)		11	(34.4)	9	(33.3)		22	(26.5)	41	(48.2)	
pT2, no. (%)	66	(57.4)	43	(38.4)		19	(59.4)	15	(55.6)		47	(56.6)	28	(32.9)	
pT3/4, no. (%)	16	(13.9)	19	(17.0)		2	(6.3)	3	(11.1)		14	(16.9)	16	(18.8)	
Estrogen receptor status (*N* = 196)					0.769^e^					0.265^e^					0.228^e^
Positive, no. (%)	61	(61.0)	61	(63.5)		18	(66.7)	12	(50)		43	(58.9)	49	(69.0)	
Negative, no. (%)	39	(39.0)	35	(36.5)		9	(33.3)	12	(50)		30	(41.1)	22	(31.0)	
Progesterone receptor status (*N* = 197)					0.776^e^					0.579^e^					1.000^e^
Positive, no. (%)	51	(50.5)	51	(53.1)		12	(44.4)	13	(54.2)		39	(52.7)	38	(53.5)	
Negative, no. (%)	50	(49.5)	45	(46.9)		15	(55.6)	11	(45.8)		35	(47.3)	33	(46.5)	

^a^High and low *BRE* expression is defined as expression above or below the median expression of the total cohort, respectively

^b^As data on radiotherapy treatment was lacking for one patient (showing high *BRE* expression), the patient numbers in the radiotherapy-treated and non-treated groups do not add up to the total cohort

^c^pT1: tumor size ≤2 cm, pT2: tumor size of 2–5 cm, pT3/4 tumor size >5 cm and/or direct extension to chest wall or skin

^d^ *p*-value is based on Mann–Whitney *U* test

^e^ *p*-value is based on χ^2^ test

^f^ *p*-value is based on Fisher Exact test

*NA* not applicable

### *BRE* QPCR

Tissue collection, mRNA isolation, and cDNA preparation have been described before [29]. *BRE* expression was measured in both cohorts by QPCR using a commercially available primer/probe set (Hs01046283_m1, Life Technologies, Carlsbad, CA, USA) and normalized to expression of the housekeeping gene *PBGD*, as described in [26]. Normalized QPCR data were mean centered per analyzed cohort and afterward the data of the cohorts were combined to increase patient numbers for further analyses.

### Statistical analyses

To statistically test the correlation of *BRE* expression with clinical parameters, the complete cohort was subdivided into two equally sized groups based on *BRE* expression. Differences in patient characteristics were tested by χ^2^, Fisher exact, or Mann–Whitney U tests, as indicated. Disease-free survival (DFS; defined as time between surgery and diagnosis of recurrent or metastatic disease) and overall survival (OS; defined as time between surgery and death by any cause) were used as feature for disease outcome. The prognostic impact of *BRE* expression was visualized by Kaplan–Meier plots and statistically tested via the logrank method and univariate or multivariate Cox regression analyses. Statistical analyses were carried out by means of Graphpad (La Jolla, CA, USA) or SPSS (IBM Corporation, Armonk, NY, USA) software.

## Results

### *BRE* expression correlates with tumor size

**Fig. 1:**
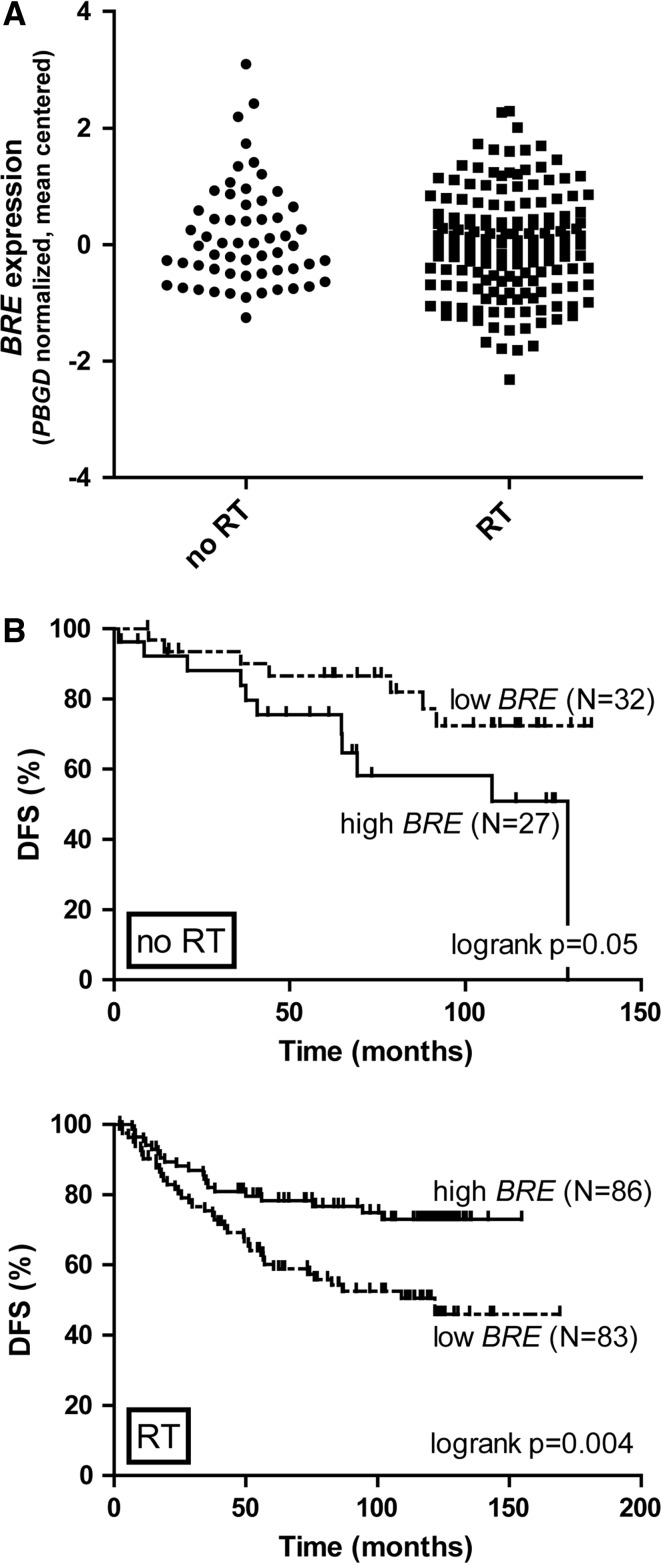
*BRE* expression predicts DFS in breast cancer. **a** *BRE* expression was gradually distributed among 229 breast cancer patients. No significant differences were observed between radiotherapy- and non-radiotherapy-treated patients. *BRE* expression was measured by QPCR and normalized with the housekeeping gene *PBGD* by calculating the ΔCt. Data shown are mean centered. Expression levels between radiotherapy-treated and non-treated patients did not differ significantly (*p* = 0.25 based on student’s *t* test). **b** For Kaplan–Meier analyses, the total cohort was divided into two equally sized groups based on *BRE* expression (high: *solid line*; low: *dashed line*, as indicated). *BRE* expression has opposing prognostic impact in non-radiotherapy-treated (no RT: *upper panel*) and radiotherapy-treated (RT: *lower panel*) patients. In non-radiotherapy-treated patients, the 5-year DFS was 86.6 ± 6.2 % and 75.5 ± 8.7 % for low and high *BRE* expression, respectively (HR = 2.59, CI = 1.00–6.75, *p* = 0.05). In radiotherapy-treated patients, the 5-year DFS was 60.2 ± 5.5 % and 78.3 ± 4.5 % for low and high *BRE* expression, respectively (HR = 0.47, CI = 0.28–0.78, *p* = 0.004). Patient numbers included in the analyses are indicated in *brackets*. *p* values, HR’s and CI’s were calculated by the logrank method. Subdividing the cohort into three groups based on *BRE* expression obtained comparable results (data not shown)

To study the prognostic effect of *BRE* expression in breast cancer, *BRE* mRNA levels were measured in tumor tissues collected at diagnosis for a cohort of 229 breast cancer patients by QPCR. Given the association of BRE with DNA damage repair, we subdivided the patient cohort a priori in two groups based on whether they had received radiotherapy as a part of their primary treatment or not. *BRE* levels were gradually distributed and no difference was observed between radiotherapy-treated or non-treated patients (*p* = 0.25). The dynamic range of expression was less than 50-fold (5.4 Ct) and levels were normally distributed (based on a Kolmogorov–Smirnov test) (see Fig. 1a). This is in contrast to AML in which *BRE* is highly expressed in a distinctive subset of patients, while the remaining patients show little variation [26].

Comparisons of *BRE* expression with known clinico-pathological factors showed that *BRE* expression correlated with tumor size (*p* = 0.014), but not with any of the other parameters (Table 1). The correlation of *BRE* expression with tumor size was only observed in radiotherapy-treated patients in which high *BRE* expression was more often found in smaller tumors (*p* = 0.005, Table 1).

### *BRE* expression predicts DFS in breast cancer

Gradual differences in *BRE* expression (using continuous QPCR data) did not correlate with DFS or overall survival (OS) in the total cohort, as tested by univariate Cox regression analysis (DFS: Table 2, OS: data not shown). However, when the cohort was subdivided into radiotherapy-treated and non-treated patients, *BRE* expression (tested as continuous variable) had prognostic impact on DFS within both groups (Table 2). Remarkably, *BRE* expression showed opposite effects on prognosis. In the radiotherapy-treated group (*N* = 169), which accounted for the majority of the patients, high *BRE* expression correlated with a favorable DFS (Hazard ratio (HR) = 0.72, 95 % confidence interval (CI) = 0.53–0.97, *p* = 0.030), while in the non-radiotherapy-treated group (*N* = 59), high *BRE* expression correlated with a poor prognosis (HR = 1.79, CI = 1.11–2.87, *p* = 0.016). Similar results were obtained when subdividing patients into two or three groups based on *BRE* expression, instead of using gradual QPCR data (Table 3, and data not shown).

**Table 2:** Univariate analysis of *BRE* expression in correlation with DFS

	Total cohort		Non-radiotherapy-treated patients		Radiotherapy-treated patients	
	*p*	HR (95 % CI)	*p*	HR (95 % CI)	*p*	HR (95 % CI)
*BRE* expression (QPCR data)	0.342	0.877 (0.67–1.15)	0.016	1.79 (1.11–2.87)	0.030	0.72 (0.53–0.97)

*HR* hazard ratio; *CI* confidence interval

**Table 3:** Multivariate Cox regression analysis of *BRE* expression correlation with DFS

	Non-radiotherapy-treated patients				Radiotherapy-treated patients			
	Univariate		Multivariate^a^		Univariate		Multivariate^a^	
	*p*	HR (95 % CI)	*p*	HR (95 % CI)	*p*	HR (95 % CI)	*p*	HR (95 % CI)
*BRE*	0.059^e^	2.51	0.083^e^	2.38	0.004	0.46	0.021	0.50
(2 groups^b^)		(0.97–6.53)		(0.89–6.35)		(0.27–0.79)		(0.27–0.90)
Age	0.349	0.98	0.616	0.99	0.112	0.98	0.020	0.97
(continuous)		(0.95–1.02)		(0.95–1.03)		(0.96–1.00)		(0.95–1.00)
Menopausal status	0.838	1.07			0.140	0.81		
(post- *vs*. premenopausal)		(0.57–1.99)				(0.62–1.07)		
Tumor size^c^	0.422	1.39	0.465	1.56	<0.001	2.01	0.014	1.70
(pT1 *vs*. pT2 *vs*. pT3/4)		(0.62–3.09)		(0.47–5.15)		(1.42–2.84)		(1.11–2.59)
Histological grade	0.941	0.97	0.895	1.01	0.032	1.70	0.313	0.95
(I *vs*. II *vs*. III *vs*. ND^d^)		(0.39–2.42)		(0.86–1.19)		(1.05–2.74)		(0.86–1.05)
Involved lymph nodes	0.002	5.63	0.034	3.92	0.001	1.87	0.013	1.66
(0 *vs*. 1–3 *vs*. ≥ 4)		(1.84–17.3)		(1.11–13.8)		(1.30–2.68)		(1.11–2.48)
Estrogen receptor status	0.680	0.82			0.362	0.78		
(positive *vs*. negative)		(0.31–2.15)				(0.45–1.34)		
Progesterone receptor status	0.866	0.92			0.839	0.95		
(positive *vs*. negative)		(0.36–2.39)				(0.55–1.62)		

^a^Factors included in multivariate analysis: *BRE* expression, age, tumor size, histological grade, and involved lymph nodes

^b^The two groups are defined as *BRE* expression above or below the median expression of the total cohort, respectively

^c^pT1: tumor size ≤2 cm, pT2: tumor size of 2–5 cm, pT3/4: tumor size >5 cm and/or direct extension to chest wall or skin

^d^As data on histological grading were missing for a substantial number of patients, this group (*ND* not done) was included in the multivariate analyses as separate group next to histological grade I, II, or III

^e^In non-radiotherapy-treated patients, *BRE* expression lost its significance when the median expression was used to divide patients based on *BRE* expression. When subdividing patients into three groups based on *BRE* expression, *BRE* was a significant predictor for DFS in both univariate and multivariate models

*HR* hazard ratio; *CI* confidence interval

**Fig. 2:**
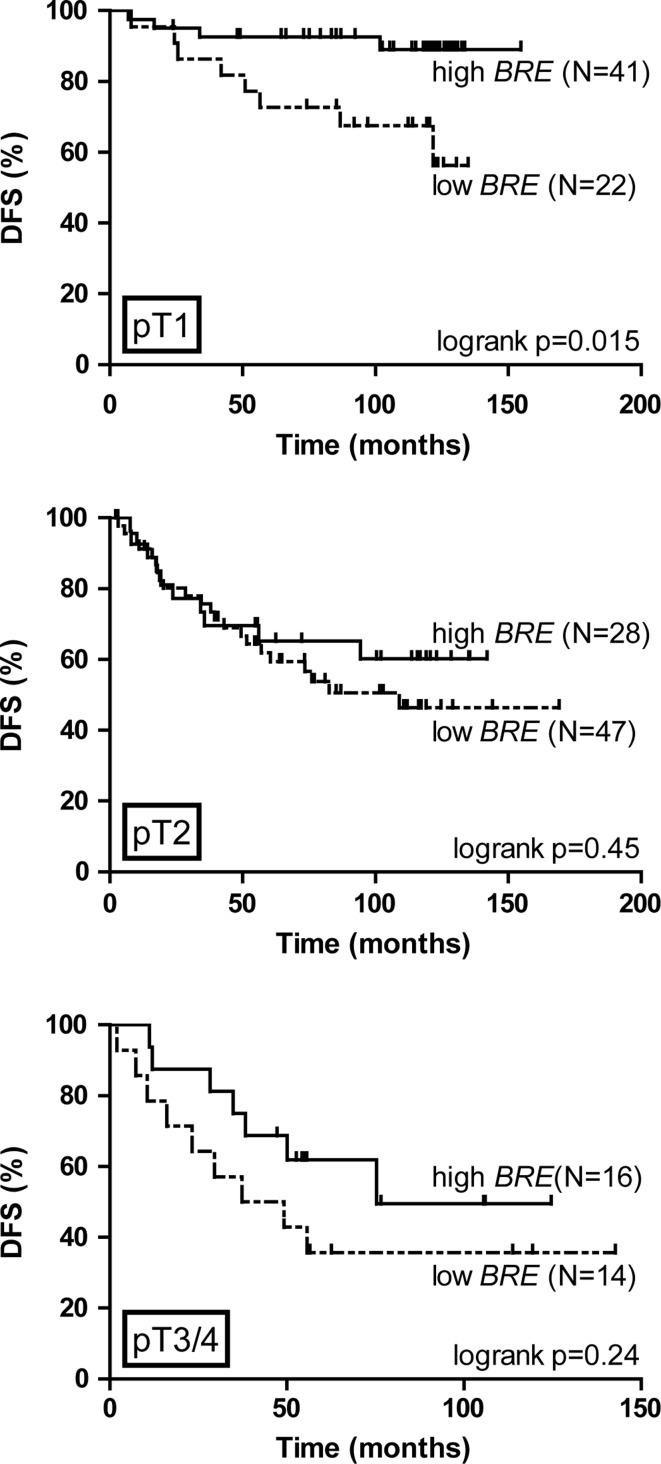
*BRE* expression predicts favorable DFS in radiotherapy-treated patients with small tumors. In radiotherapy-treated patients, *BRE* expression predicts DFS in patients with small tumors (pT1, *upper panel*). The 5-year DFS was 72.7 ± 9.5 % and 92.6 ± 4.1 % for low and high *BRE* expression, respectively (HR = 0.23, CI = 0.068–0.75, *p* = 0.015). For patients with larger tumors, no statistically significant prognostic effect of *BRE* expression was observed. For this analysis, patients were subdivided into two groups based on *BRE* expression, as explained in Fig. 1. *p*-values, HR’s, and CI’s were calculated by the logrank method

The effect of *BRE* expression on DFS was visualized by Kaplan–Meier plots by subdividing the total cohort into two groups using the median of *BRE* expression as cut-off. Among the patients who did not receive radiotherapy, high *BRE* expression predicted an adverse prognosis validating the Cox regression analysis (HR = 2.59, CI = 1.00–6.75, *p* = 0.05). High *BRE* expression predicted a favorable prognosis among the patients who received radiotherapy (HR = 0.47, CI = 0.28–0.78, *p* = 0.004) (Fig. 1b). Interestingly, within the radiotherapy-treated patients, a significant correlation between *BRE* expression and DFS was only observed for the group of patients with smaller tumors (HR = 0.23, CI = 0.068–0.75, *p* = 0. 015), which contained relatively more high *BRE* expressing patients (Table 1; Fig. 2). No significant prognostic impact was observed in patients with larger tumors (Fig. 2). Radiotherapy was combined with adjuvant systemic treatment for a part of the cohort (see Table 1). To exclude the possibility that the effect of *BRE* expression on prognosis depended on the combination of radiotherapy and adjuvant treatment, we calculated the effect of *BRE* expression on DFS for patients treated by radiotherapy only (94 of the 169 patients that received radiotherapy). This analysis showed that also within this subcohort, high *BRE* expression predicted favorable disease outcome (HR = 0.38, CI = 0.18–0.78, *p* = 0.009, data not shown). Within the group of patients who did not receive radiotherapy, 75 % did not receive adjuvant treatment either (44 of the 59 patients). Within this group of patients, the impact of *BRE* expression on DFS lost its significance (data not shown). This might indicate that in non-radiotherapy treated patients, the effect of *BRE* expression on DFS is dependent on adjuvant treatment. However, as the number of patients receiving adjuvant treatment without radiotherapy was too small, we were not able to test this hypothesis.

### *BRE* expression is an independent prognostic factor in radiotherapy-treated patients

To determine whether *BRE* expression was an independent prognostic factor for DFS in breast cancer, multivariate Cox regression analyses were performed. These analyses showed that *BRE* expression was a prognostic factor within the group of radiotherapy-treated patients, independent of other tested prognostic factors such as age, tumor size, lymph node involvement, and histological grade (HR = 0.50, CI = 0.27–0.90, *p* = 0.021, shown in Table 3). Of note, also age, tumor size, and the number of involved lymph nodes were independent prognostic factors in this group of patients. For non-radiotherapy-treated patients, *BRE* expression did not correlate significantly with DFS in the multivariate analysis.

### *BRE* expression predicts outcome in a large independent breast cancer cohort

**Fig. 3:**
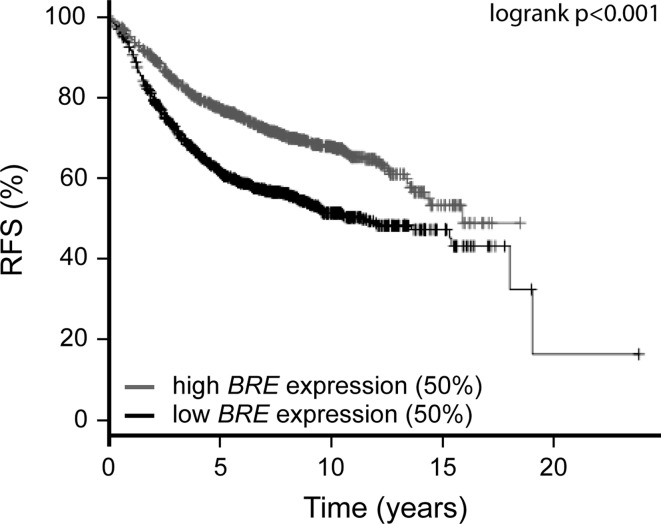
*BRE* expression predicts relapse-free survival in a cohort of 2,324 breast cancer patients. A publicly available database (Kaplan–Meier Plotter [32]) was used to investigate the effect of *BRE* expression on relapse-free survival (RFS) in a cohort of 2,324 breast cancer patients. Array data (probe set 211566_s_at) of these patients were used to divide patients into two equally sized groups. High *BRE* expression predicts a favorable prognosis (HR = 0.51, CI = 0.51–0.68, *p* < 0.001). *p*-value, HR, and CI were calculated by the logrank method

To determine whether *BRE* expression has an impact on survival in other patient cohorts, we extended our studies to a large independent, publicly available micro-array dataset of 2,324 patients (see Fig. 3, Kaplan–Meier Plotter [32] (www.kmplot.com)). We observed a favorable prognosis for patients with high *BRE* expression (upper 50 % of the patients) and an adverse survival for patients with low *BRE* expression (lower 50 %) (HR = 0.59, CI = 0.51–0.68, *p* < 0.001 after correction for multiple testing). The data of this cohort resembled the data of the first cohort of radiotherapy-treated patients (Fig. 1b). However, as no data were available on the number of patients who received radiotherapy within this publically available cohort, we were unable to test whether the prognostic effect of *BRE* expression was influenced by radiotherapy treatment.

## Discussion

High expression of the BRCA1 complex member BRE has recently been identified in a subgroup of AML patients in whom it defines favorable prognosis [25, 26]. Here, we show that the expression of this gene also predicted disease outcome in a cohort of 229 non-familial breast cancer patients. Interestingly, the predictive value of *BRE* expression at diagnosis on DFS depended on whether the patient received subsequent radiotherapy treatment or not. In radiotherapy-treated patients, high *BRE* expression predicted a favorable disease outcome, whereas in non-radiotherapy-treated patients, it correlated with an adverse outcome (see Fig. 1; Table 3). To extend our studies, *BRE* expression was evaluated in a publicly available dataset of 2,324 breast cancer patients [32]. In this large cohort, high *BRE* expression predicted a favorable relapse-free survival, resembling the data of radiotherapy-treated patients within the cohort studied in this manuscript (Fig. 1b). The large cohort of 2,324 patients represents a collection of previously published gene expression datasets, for which integral data on clinico-pathological factors are unavailable. Therefore, we were unable to determine the impact of radiotherapy on the effect of *BRE* expression on disease outcome in this cohort. The identification of prognostic impact of *BRE* expression in two independent cohorts warrants further studies in large cohorts to validate the effects found in radiotherapy-treated and non-treated patients.

The fact that *BRE* expression predicted opposing effects on disease outcomes depending on radiotherapy treatment might imply that there are intrinsic differences in breast cancer patients who are treated or not treated with radiotherapy. Alternatively, there might be a direct effect of high *BRE* expression on radiotherapy response. The effect of *BRE* expression on disease outcome was not due to co-treatment with adjuvant therapy within the radiotherapy-treated group of patients as the effect of *BRE* expression on DFS was also present in the subgroup of radiotherapy-treated patients who did not receive adjuvant treatment. The decision for radiotherapy treatment is closely related to surgical treatment and depends on multiple factors like tumor size and the involvement of axillary lymph nodes. As the patients were consequently not randomly assigned for treatment, it was not possible to explain the opposing effect of *BRE* expression on the prognosis of radiotherapy-treated versus non-treated patients in this cohort. Therefore, it would be of particular interest to test *BRE* expression in a cohort of patients who received radiotherapy in a randomized fashion to evaluate a direct effect of *BRE* expression on therapy outcome.

BRE is a member of the BRCA1 complex involved in DNA double strand break repair [21–24]. This complex is recruited to DNA damaged sites via binding of the complex member Rap80 to ubiquitin chains, which are generated upon DNA damage [33–35]. Mutations in DNA damage repair factors are closely linked to familial breast cancer as 25 % of these cases is characterized by mutations in factors involved in the DNA damage repair pathway, like BRCA1, BRCA2, PTEN, p53, CHEK2, and ATM [7–9, 36–39]. However, in non-familial breast cancer, these mutations are rare. In non-familial cases, associations between low BRCA1 expressions with poor prognosis have been identified [16–19] resembling the observations we made for *BRE* expression in radiotherapy-treated patients.

Depletion of BRE abrogates BRCA1 foci formation, indicating that BRE is needed for complex formation and downstream DNA repair [22–24, 40]. Several studies have described an increased radiosensitivity of cells after *BRE* depletion [21, 22]. Next to a role in the BRCA1 complex, BRE is also involved in death receptor-mediated apoptosis as it binds TNFα and FAS receptors, and overexpression of *BRE* caused resistance to apoptosis induction by various stress-related stimuli [41]. This indicates that BRE serves an anti-apoptotic role following different types of stress. It was therefore unexpected to find a positive correlation between high *BRE* expression and breast cancer outcome in relation to radiotherapy. High expression would enhance DNA repair and hence would render cells resistant to radiotherapy. Indeed, this reasoning seems to be true for BRCA1 as radiotherapy has been shown to be especially beneficial for patients with low BRCA1 levels, whereas there was no benefit for patients with high BRCA1 levels [42]. On the other hand, high expression of the Mre11/Rad50/Nbs1 complex, also involved in DNA damage repair, predicts a good response to radiotherapy [43], indicating that DNA repair proteins can contribute differentially to radiotherapy response. In this case, high *BRE* expression might attenuate the DNA damage repair pathway following radiotherapy. Potentially, high *BRE* expression causes a misbalance in the BRCA1 multi-protein complex formation, thereby reducing the functionality of the complex and rendering cells more sensitive to radiation-induced DNA damage. It would be of particular interest to study the subcellular localization of *BRE* in these tumors to determine whether responses can be attributed to the DNA damage response or death receptor signaling. The data described in this study indicate that *BRE* is an interesting candidate for further functional studies in breast cancer to test its effect on radiotherapy responses.
